# An unusual case of a glottic carcinoma metastasis to the tracheal lumen

**DOI:** 10.3332/ecancer.2018.846

**Published:** 2018-07-03

**Authors:** Sarantis Blioskas, Peter Karkos, Iordanis Konstantinidis, Kyriakos Chatzopoulos, George Psillas, Prodromos Chytiroglou, Konstantinos Markou

**Affiliations:** 1 Department of Otorhinolaryngology—Head and Neck Surgery, 424 Military Hospital of Thessaloniki, 54629 Thessaloniki, Greece; 2 1st Department of Otorhinolaryngology—Head and Neck Surgery, Aristotle University of Thessaloniki, AHEPA Hospital, 54636 Thessaloniki, Greece; 3 2nd Department of Otorhinolaryngology—Head and Neck Surgery, Aristotle University of Thessaloniki, Papageorgiou Hospital, 54629 Thessaloniki, Greece; 4 Department of Pathology, Aristotle University of Thessaloniki, AHEPA Hospital, 54636 Thessaloniki, Greece

**Keywords:** glottic cancer, endotracheal, metastasis, lymphatic spread

## Abstract

Various mechanisms such as second primary lesion, tumour seeding or lymphogenous and haematogenous metastasis could be proposed to explain the nature of dual malignant lesions. We report the case of a glottic laryngeal carcinoma combined with a secondary endotracheal tumour. Following the imaging modalities, the patient underwent total laryngectomy and wide excision of the trachea. Histopathology ultimately established that the tracheal lesion was a metastatic tumour secondary to regional lymphatic spread of the glottic tumour. To our knowledge, there is no previous report in the English literature concerning tracheal lymphogenous metastatic involvement in the context of laryngeal malignancy. Paradoxical lymphatic spread must always remain an issue of head and neck oncology.

## Introduction

Laryngeal cancer commonly metastasizes via the cervical lymph nodes to distant sites. Haematogenous spread occurs less frequently, accounting for only about 10% of all distant metastases. The most common site for laryngeal squamous cell carcinoma metastasis is the lungs, followed by the liver, bones and central nervous system [1]. Yet, to the best of our knowledge, tracheal metastatic tumours have not been reported in the English literature.

We report the case of a glottic laryngeal carcinoma with solitary endotracheal metastasis.

## Case report

A 60-year-old male was referred to our department, complaining about gradually worsening hoarseness, during the last 8 month period. Occasional dysphagia and foreign-body sensation were also reported upon referral. The patient was a heavy smoker for more than 20 years, reporting an average of 20 cigarettes per day. Alcohol was also a factor, and although no real alcohol abuse or indulgence was noted, the patient was a rather frequent user.

Medical history only revealed arterial hypertension under treatment with beta blockers. Haematological and biochemical tests did not show any significant abnormalities.

Physical examination included a full head and neck examination, complemented with flexible fiberoptic laryngoscopy. Typical ear, nose and throat examination did not reveal any abnormal findings and neck palpation was negative. However, fiberoptic laryngoscopy revealed a lesion affecting both vocal cords and anterior commissure, while vocal cord mobility appeared impaired. On these grounds, a cervicothoracic and upper abdomen computed tomography (CT) scan with intravenous gadolinium was decided and the patient was scheduled for direct microlaryngoscopy and biopsy of the lesion under general anaesthesia.

**Figure 1.**
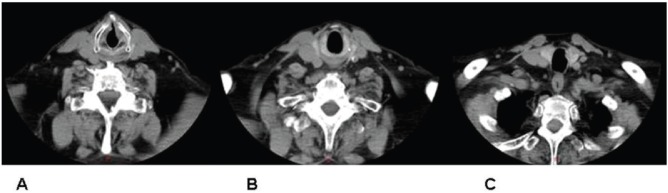
CT indicating both glottic (A) and tracheal (C) tumours. Intermediate tissue appears grossly normal (B).

Imaging confirmed the laryngeal lesion, yet it also indicated a second lesion about 2 cm below the inferior end of the primary one, arising somewhere between the first and second tracheal ring. Intermediate tissue appeared grossly normal (Figure 1). No signs of enlarged cervical lymph nodes were noted and laryngeal cartilages showed no abnormal findings.

On the other hand, histopathological examination after biopsy of the lesion under general anaesthesia confirmed the diagnosis of squamous cell carcinoma. The lesion was carefully mapped and proved to be a glottic carcinoma affecting the anterior commissure and appearing in strong correlation with the thyroid cartilage. The lesion infiltrated the left and the first tertile of the right vocal cord. No subglottic extension was noted. In this context, the patient was informed and consent for radical surgical therapy was obtained.

**Figure 2.**
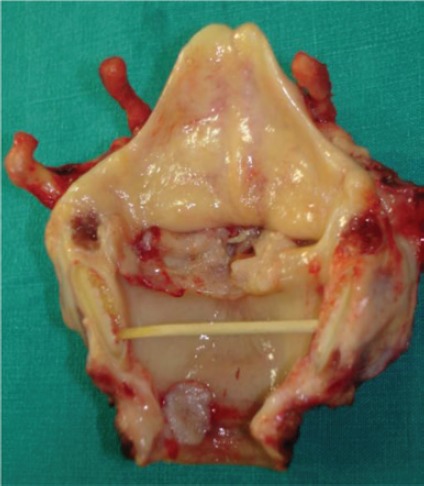
Surgical specimen verifies the presence of both a glottic and an endoluminal tracheal tumour. Intermediate tissue shows no macroscopic signs of malignancy.

The patient underwent total laryngectomy and wide excision of the trachea which included the second tumour within safe limits (Figure 2). The procedure was complimented with left thyroid lobectomy and bilateral selective neck dissection (Robin’s levels II–IV). Paratracheal lymph nodes (Robin’s level VI) were also carefully dissected. The overall postoperative course was uneventful. The patient was discharged from our department on day 16 with very good swallow function and was decannulated after 1 week. Surgical resection was followed by postoperative radiation therapy (6400 cGy/32 fraction).

**Figure 3.**
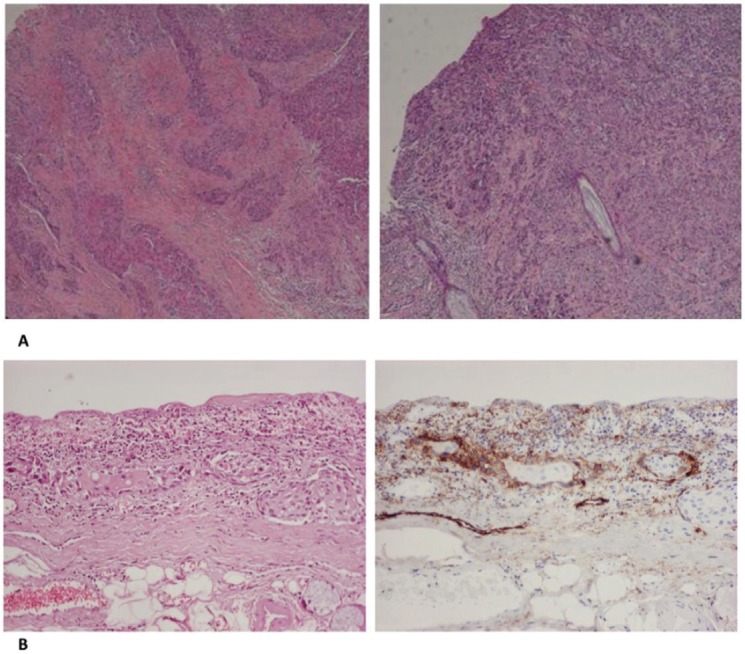
(a): The tumour of the glottis (left) and the trachea (right) share the same morphological features. (b): Haematoxylin-eosin stain shows groups of tumour cells in lymphatic vessels (left). Immunοhistochemical stain for D2-40 antigen shows positivity of the vascular endothelium, confirming that these are indeed lymphatics (right). (B: ×200).

The final pathological report was of crucial importance in our case. First of all, the surgical margins of resection were found to be free of disease. Second, histological sections from the tumour of the glottis showed the characteristic morphology of squamous cell carcinoma. Cancer cells were large in size and polygonal in shape with eosinophilic cytoplasm and nuclei with moderate variation in size and shape. There were a moderate number of mitoses and keratinisation could be focally observed. Cancer cells showed an infiltrative pattern consisting mainly of nests and trabeculae that invaded the vocalis muscle in both the vocal cords. The perichondrium of thyroid cartilage was focally invaded by cancer cells. Histological sections from the tumour of the trachea showed morphological features identical to those of the tumour of the glottis. An upward infiltrating pattern could be noticed. Moreover, a comparative immunohistochemical study of the two tumours showed strong positivity of cancer cells in stains for keratins AE1/AE3 and 34βΕ12 and moderate positivity in stains for CK5/6, CK8/18 and epithelial membrane antigen. Immunohistochemistry for D2-40 antigen (podoplanin) illustrated the positivity of the lymphatic endothelium. Immunohistochemical stains for other vascular endothelia (CD31 and CD34 antigens) were also performed, and were negative. In the region between the two tumours, many lymphatics containing neoplastic emboli could be observed (Figure 3). Finally, two tumour-infiltrated lymph nodes (the larger being of 1.2 cm diameter) with extracapsular spread were found in the left neck dissection specimen. A pT4a(m)N2b stage, according to eighth edition TNM staging, was established.

## Discussion

Various mechanisms could be proposed to explain the nature of such a rare case of dual lesions.

The case of a simultaneous second primary tracheal tumour must be accounted for. First of all, in order to clarify terminology, we use the established term ‘simultaneous’ to refer to primary tumours diagnosed at the same time, whereas ‘synchronous’ refers to lesions detected within a 6-month [2] or 1 year [1] period of identification of the index tumour. On these grounds, although tumours at hand are in fact simultaneous, it remains a fact that the patient did not seek medical care 8 months before the initial symptomatology and no imaging of the area was for any reason undertaken prior to that. Thus, the possibility of the tracheal tumour being a synchronous lesion of primary (or even metastastic) nature cannot be excluded relying simply on the concurrent diagnosis.

Multicentric carcinomas of the aerodigestive tract are not rare. The literature suggests that the patients with primary squamous cell carcinoma in the upper aerodigestive tract run an increased risk of developing a second tumour [3]. The concept of ‘field cancerisation’ or ‘condemned mucosa syndrome’ [4] suggesting elevated epithelial cancer risk of a field of mucosa afflicted with environmental carcinogenic agents, is well established. Yet, accepted criteria [5] would not characterise a lesion of the same histology and region as second primary tumour, unless developed 60 months after index diagnosis. In addition, large series published studies [2] have not reported the trachea as a possible site for second primary tumours and an extensive literature review revealed no reported cases of multicentric malignancy involving both the larynx and trachea. Ultimately, morphological and immunohistochemical identity of both lesions indicated in the permanent pathological report does not preclude the possibility of two separate primary tumours, yet yields tentative evidence for the opposite.

Moreover, the possibility of neoplastic spread by tumour cell seeding would be an alternative worth taking into consideration. Neoplastic seeding is a well-documented concept of mechanical tumour disruption and consequent cell implantation into adjacent or distant traumatised tissues. Although it remains a rare mechanism of tumour dissemination, yet it is accepted as an explanation for unusual patterns of tumour recurrence. Tumour implantation into tracheotomy and percutaneous endoscopic gastrostomy sites has been postulated [6, 7] in patients with head and neck tumours, without excluding rarer locations [8]. Particularly, in tracheostomy site spreading, mechanical implantations usually take place by intraluminal contamination, through aspiration of cancer cells, thus exposing the tracheal lumen into the danger of neoplastic seeding. Likewise, in our case even intubation alone could potentially traumatise the tissue in the area where the second tumour evolved and consequently disseminate neoplastic cells from the glottis to damaged tissue and promote seeding. However, our patient did not report any major surgery in the area at hand or any prior intubation, thus such a mechanism is highly unlikely.

Finally, the possibility of a metastatic endoluminal tracheal tumour was considered. Lymphogenous and haematogenous metastasis of laryngeal cancer are not rare. The lymphatic metastatic pattern of laryngeal squamous cell carcinoma is well known. Nodal metastasis is mainly concentrated in cervical regional lymph nodes, particularly Robin’s levels IIa and III while metastases outside of this level are rather unusual [9]. Advanced regional nodal disease increases the incidence of distant metastases threefold [1]. Distant metastasis is defined as tumour spread to other organ systems either haematogenous or lymphatic to other than regional lymph nodes [1]. Reported clinical incidence of distant metastases in head neck malignancies appears to be between 11% and 24%, while autopsy studies demonstrate a much higher incidence [1]. The lung, liver and bone are the commonest sites for haematogenous tumour spread, whereas mediastinum appears to be the most common lymphatic distant metastatic site [1]. Yet, to our knowledge, no haematogenous or lymphatic metastasis of a laryngeal carcinoma to the trachea has been reported, thus doubts were initially raised.

The histopathology report shed light on these key questions, although an effort to make a molecular comparison of the two tumours was unsuccessful, due to insufficient DNA preservation. The identical morphological and immunohistochemical features of the two tumours and the upward infiltrating pattern of the tracheal tumour, combined with the striking finding of neoplastic emboli in intermediate lymphatics were not decisive about the nature of the lesions, however, established that against all odds the tracheal lesion was most probably a metastatic tumour secondary to regional lymphatic spread of the tumour of the glottis. Since the tracheal lumen has not been reported as a possible metastatic site for glottic carcinomas, this unique case underlines the need for all head and neck surgeons to remain vigilant for paradoxical lymphatic tumour spread.

## Conclusion

We report a unique case of endoluminal tracheal metastasis of a glottic carcinoma, secondary to regional lymphatic spread. To our knowledge, there is no previous report in the English literature concerning tracheal metastatic involvement in the context of laryngeal malignancy. Paradoxical lymphatic spread must always remain an issue within the field of head and neck oncology.

## Conflicts of interest

The author(s) declare that they have no conflict of interest.
